# Supplementary material for: Controlled Release in Hydrogels Using DNA Nanotechnology
Source: Biomedicines. 2022 Jan 19;10(2):213. doi: 10.3390/biomedicines10020213 (PMC8869372; doi:10.3390/biomedicines10020213)
Supplement: Supplementary file 1 [file biomedicines-10-00213-s001.zip › biomedicines-1545456-supplementary.pdf]

Article

Controlled Release in Hydrogels Using DNA Nanotechnology

Chih-Hsiang Hu and Remi Veneziano *

Department of Bioengineering, College of Engineering and Computing, George Mason University, Manassas, VA 20110, USA; chu6@gmu.edu
* Correspondence: rvenezia@gmu.edu

Table S1. List of DNA sequences used in this study. Sequences are from 5′ to 3′.

Base Design	
**Name**	**Sequence**
3WJ-1	CTGACCGAGCGTGGCTACAGCTTC TTT GGTTCACCGCTTGCCTTCTGCTCT CGCTGTGGCACCTGCACG
3WJ-2	CCTGCTTGCTGATCCACATCTGCT TTT GAAGCTGTAGCCACGCTCGGTCAG GCATCGGGTATCCAGTGG
3WJ-3	AGAGCAGAAGGCAAGCGGTGAACC TTT AGCAGATGTGGATCAGCAAGCAGG TACGGTCGGCGCTGTATG
4WJ-3	AGAGCAGAAGGCAAGCGGTGAACC TTT CGTGACTGCACCTGAATTGGCACT TGCGCCATCCCTTCAGAG
4WJ-4	AGTGCCAATTCAGGTGCAGTCACG TTT AGCAGATGTGGATCAGCAAGCAGG TACGGTCGGCGCTGTATG
DNA Bait	/Azide/ TTT CGTGCAGGTGCCACAGCG
Trigger 1	CATAACACA TCT CACAATCCA TCT CACCACCCA
Payload 1 Top	CACAATCCA TCT CACCACCCA /Iowa Black FQ/
Payload 1 Bottom	/HEX/ TGGGTGGTG AGA TGGATTGTG AGA TGTG AGA CATACAGCGCCGACCGTA
Trigger 2	CACAATCCA TCT CACCACCCA TCT CAAAACTCA
Payload 2 Top	CACCACCCA TCT CAAAACTCA /Iowa Black FQ/
Payload 2 Bottom	/FAM/ TGAGTTTTG AGA TGGGTGGTG AGA TGGA AGA CCACTGGATACCCGATGC
Trigger 3	CACCACCCA TCT CAAAACTCA TCT CATCCAACA
Payload 3 Top	CAAAACTCA TCT CATCCAACA /Iowa Black SQ/
Payload 3 Bottom	/Texas-Red/ TGTTGGATG AGA TGAGTTTTG AGA TGGG AGA CTCTGAAGGGATGGCGCA
Bait Probe	/HEX/ TTT CGCTGTGGCACCTGCACG
**Mismatched Triggers**	
**Name**	**Sequence**
1bp-center (TrigA)	CATAACACA TCT CACA T TCCA TCT CACCACCCA
2bp-center (TrigB)	CATAACACA TCT CACA TG CCA TCT CACCACCCA
3bp-center (TrigC)	CATAACACA TCT CAC TTG CCA TCT CACCACCCA

5bp-center (TrigD)	CATAACACA TCT CA ATTGG CA TCT CACCACCCA
1bp-3′ end (TrigE)	CATAACACA TCT CACAATCCA TCT CACC G CCCA
2bp-3′ end (TrigF)	CATAACACA TCT CACAATCCA TCT CACC GG CCA
3bp-3′ end (TrigG)	CATAACACA TCT CACAATCCA TCT CAC AGG CCA
5bp-3′ end (TrigH)	CATAACACA TCT CACAATCCA TCT CA AAGGT CA

Table S2. Individual measurements of gelatin modification quantification via fluorescamine.

Theoretical [DBCO] (µM)	Measured [DBCO] (µM)	Efficiency
**135**	142.43	106%
**135**	78.87	58%
**135**	74.83	55%
**135**	113.09	84%
**135**	122.14	90%
**135**	166.90	124%

Table S3. Individual measurements of DNA overhang quantification via overhang probe.

Theoretical [DNA Overhang] (µM)	Measured [DNA Overhang] (µM)	Efficiency
**45**	20.71	46%
**45**	31.06	69%
**45**	42.78	95%
**45**	19.14	43%
**45**	21.49	48%

Table S4. 50% Release Time (**T_{50}**) for 4WJ.

4WJ Type	Measured T_{50} (mins)	Fitted T_{50} (mins)
**HEX**	9.5	9.71
**FAM**	11.7	12.01
**TR**	10.6	9.69

Table S5. Dunn's Kruskal-Wallis Multiple Comparison *post-hoc* analysis table for mismatch trigger release, which corresponds to Figure 3d,f in the main text.

No.	Comparison	Z Score	p Value
**1**	4WJ_HEX_MT1_13' - 4WJ_HEX_MT1_1C	1.2396648	0.2150994
**2**	4WJ_HEX_MT1_13' - 4WJ_HEX_MT1_23'	1.1986678	0.2306572
**3**	4WJ_HEX_MT1_1C - 4WJ_HEX_MT1_23'	-0.102012	0.9187471
**4**	4WJ_HEX_MT1_13' - 4WJ_HEX_MT1_2C	2.5746262	0.01003485
**5**	4WJ_HEX_MT1_1C - 4WJ_HEX_MT1_2C	1.2082823	0.2269387
**6**	4WJ_HEX_MT1_23' - 4WJ_HEX_MT1_2C	1.3862501	0.1656706
**7**	4WJ_HEX_MT1_13' - 4WJ_HEX_MT1_33'	3.421316	0.000623189
**8**	4WJ_HEX_MT1_1C - 4WJ_HEX_MT1_33'	2.058649	0.03952788
**9**	4WJ_HEX_MT1_23' - 4WJ_HEX_MT1_33'	2.2775367	0.0227542
**10**	4WJ_HEX_MT1_2C - 4WJ_HEX_MT1_33'	0.9402715	0.3470783
**11**	4WJ_HEX_MT1_13' - 4WJ_HEX_MT1_3C	4.4411638	8.94737E-06
**12**	4WJ_HEX_MT1_1C - 4WJ_HEX_MT1_3C	3.0207058	0.002521862
**13**	4WJ_HEX_MT1_23' - 4WJ_HEX_MT1_3C	3.3008608	0.000963887
**14**	4WJ_HEX_MT1_2C - 4WJ_HEX_MT1_3C	1.9576411	0.05027214
**15**	4WJ_HEX_MT1_33' - 4WJ_HEX_MT1_3C	0.9743393	0.3298881
**16**	4WJ_HEX_MT1_13' - 4WJ_HEX_MT1_53	5.4375361	5.40224E-08
**17**	4WJ_HEX_MT1_1C - 4WJ_HEX_MT1_53	3.9881917	6.65789E-05
**18**	4WJ_HEX_MT1_23' - 4WJ_HEX_MT1_53	4.3228951	1.53995E-05
**19**	4WJ_HEX_MT1_2C - 4WJ_HEX_MT1_53	3.0026453	0.002676442
**20**	4WJ_HEX_MT1_33' - 4WJ_HEX_MT1_53	1.9963735	0.04589328
**21**	4WJ_HEX_MT1_3C - 4WJ_HEX_MT1_53	1.0450042	0.296021
**22**	4WJ_HEX_MT1_13' - 4WJ_HEX_MT1_5C	6.2147066	5.14206E-10
**23**	4WJ_HEX_MT1_1C - 4WJ_HEX_MT1_5C	4.7428306	2.10752E-06
**24**	4WJ_HEX_MT1_23' - 4WJ_HEX_MT1_5C	5.1200818	3.05403E-07
**25**	4WJ_HEX_MT1_2C - 4WJ_HEX_MT1_5C	3.8177485	0.000134675
**26**	4WJ_HEX_MT1_33' - 4WJ_HEX_MT1_5C	2.7935602	0.005213132
**27**	4WJ_HEX_MT1_3C - 4WJ_HEX_MT1_5C	1.8601074	0.06287033
**28**	4WJ_HEX_MT1_53 - 4WJ_HEX_MT1_5C	0.8151032	0.4150132
**29**	4WJ_HEX_MT1_13' - 4WJ_HEX_T1	-0.6403353	0.5219546
**30**	4WJ_HEX_MT1_1C - 4WJ_HEX_T1	-1.913472	0.05568765
**31**	4WJ_HEX_MT1_23' - 4WJ_HEX_T1	-1.9115137	0.0559386
**32**	4WJ_HEX_MT1_2C - 4WJ_HEX_T1	-3.3718801	0.00074657
**33**	4WJ_HEX_MT1_33' - 4WJ_HEX_T1	-4.2380353	2.25484E-05
**34**	4WJ_HEX_MT1_3C - 4WJ_HEX_T1	-5.3295212	9.8472E-08
**35**	4WJ_HEX_MT1_53 - 4WJ_HEX_T1	-6.3745254	1.83531E-10
**36**	4WJ_HEX_MT1_5C - 4WJ_HEX_T1	-7.1896286	6.49678E-13

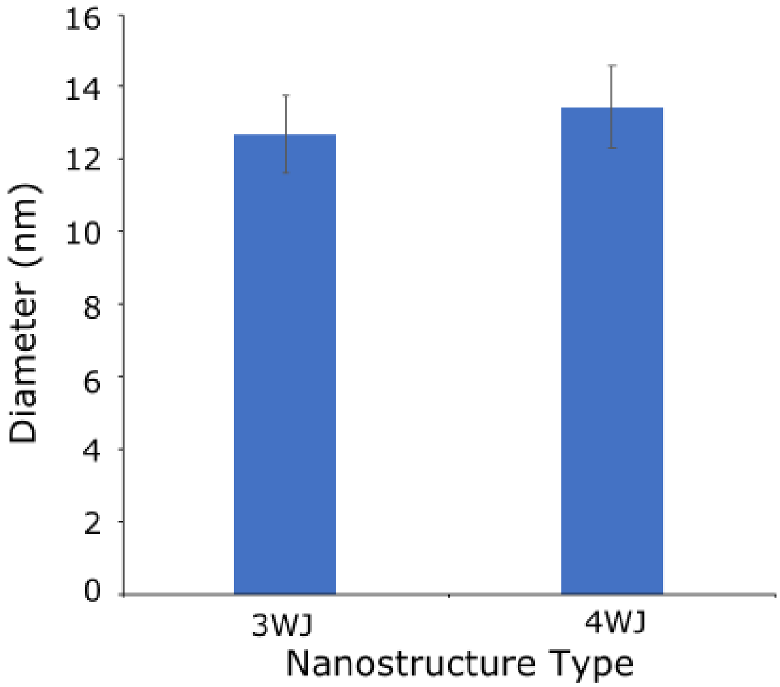

Figure S1. DLS Size Measurements for Bare 3WJ and 4WJ. (Welch two sample t-test, n = 9, $p > 0.05$).

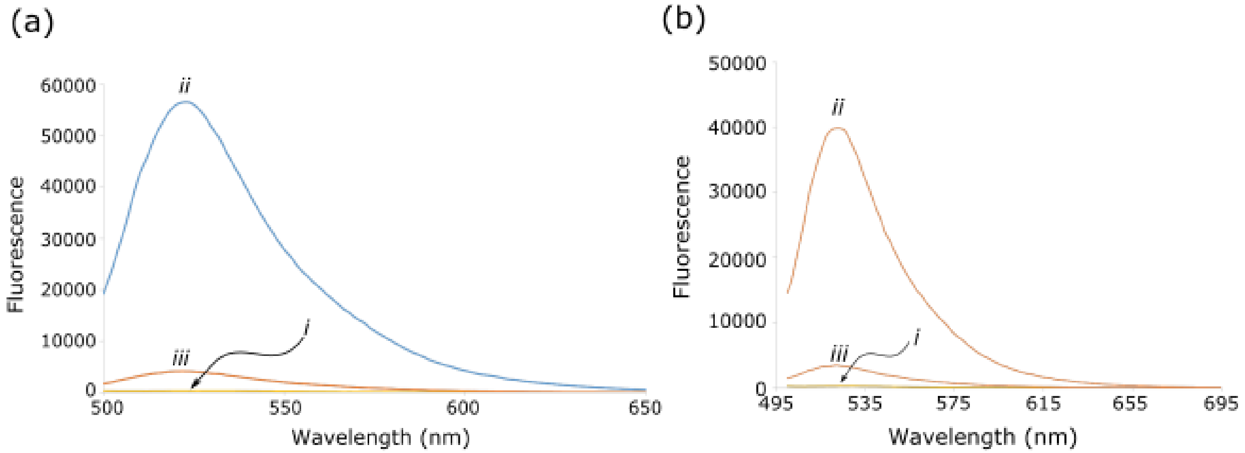

Figure S2. Fluorescent quantification of 4WJ (**a**) Fluorescent quantification of 4WJ-FAM. The excitation wavelength of FAM was 455 nm and emission spectrum was recorded from 500 nm to 800 nm. (**b**) Fluorescent quantification of 4WJ-TR. The excitation wavelength of Texas-Red was 555 nm and emission spectrum was recorded from 590 nm to 800 nm. (i) bare 4WJ (ii) 4WJ-1F (iii) 4WJ-1FQ.

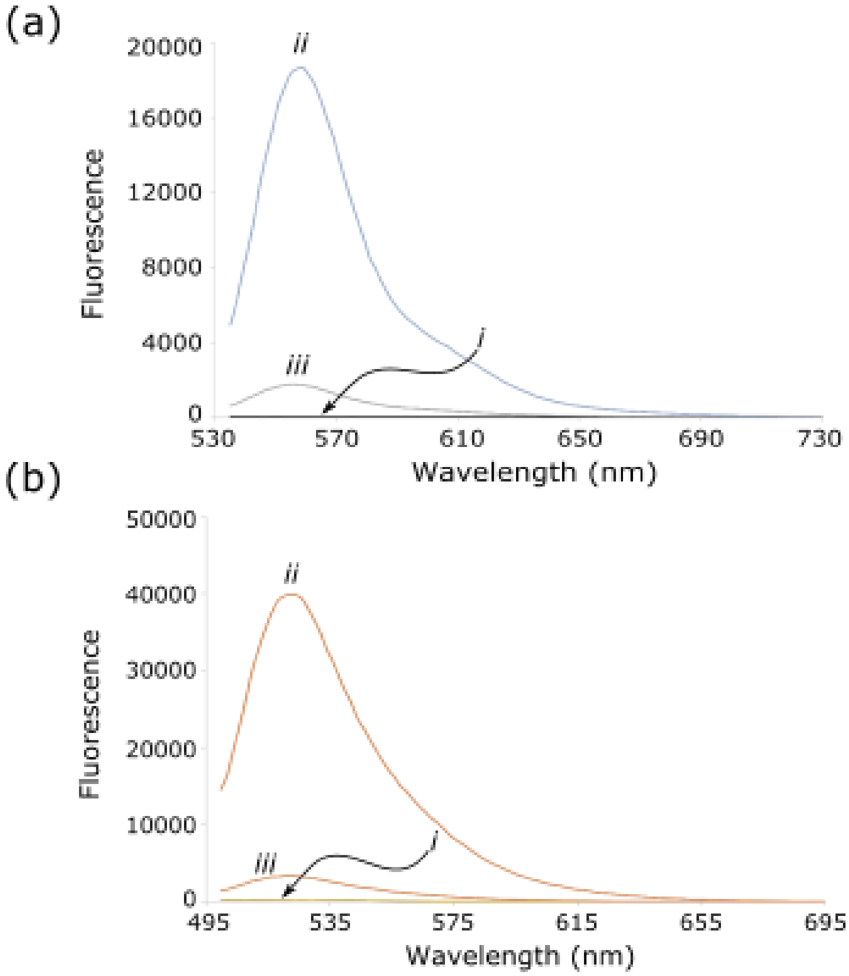

Figure S3. Fluorescent quantification of 3WJ (**a**) 3WJ-HEX. The excitation wavelength of HEX was 495 nm and emission spectrum was recorded from 535 nm to 800 nm (**b**) 3WJ-FAM. The excitation wavelength of FAM was 455 nm and emission spectrum was recorded from 500 nm to 800 nm. (i) bare 3WJ (ii) 3WJ-1F (iii) 3WJ-1FQ.

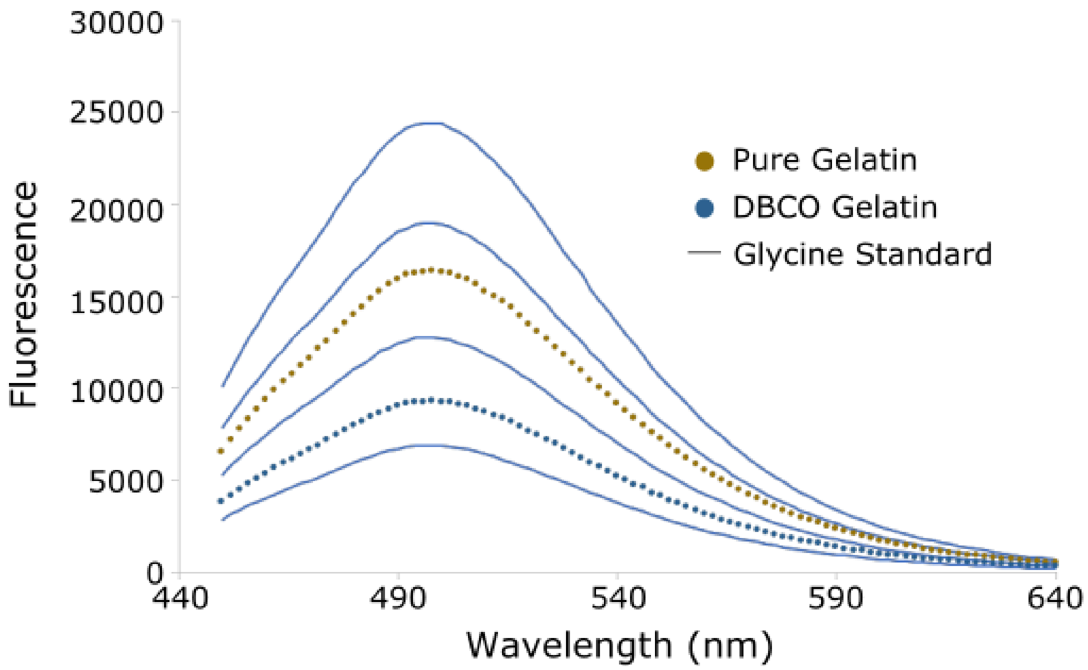

Figure S4. Representative Fluorescence Curves of Fluorescamine for Both Glycine Standard and Pure and DBCO Gelatin Samples. Standard range from 50–200 nM of Glycine. Fluorescamine was excited at a wavelength of 380 nm and the emission spectrum was recorded from 450 nm to 750 nm with step size of 2 nm.

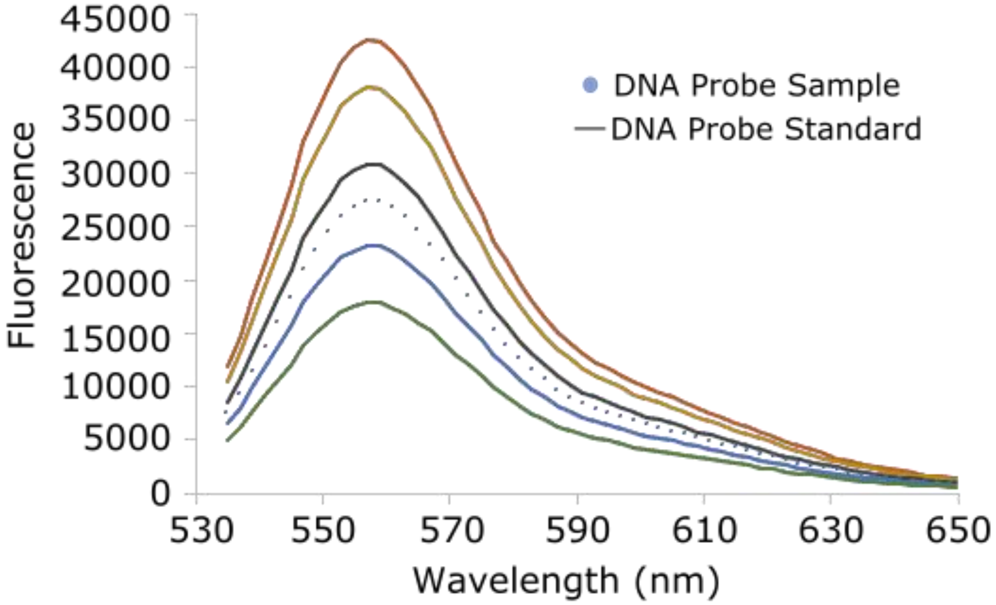

Figure S5. Representative Fluorescence Curves of DNA Bait Quantification for Both DNA Probe Standard and Sample. Standard range from 300 to 700 nM of DNA Probe. The excitation wavelength of HEX was 495 nm and emission spectrum was recorded from 535 nm to 800 nm.

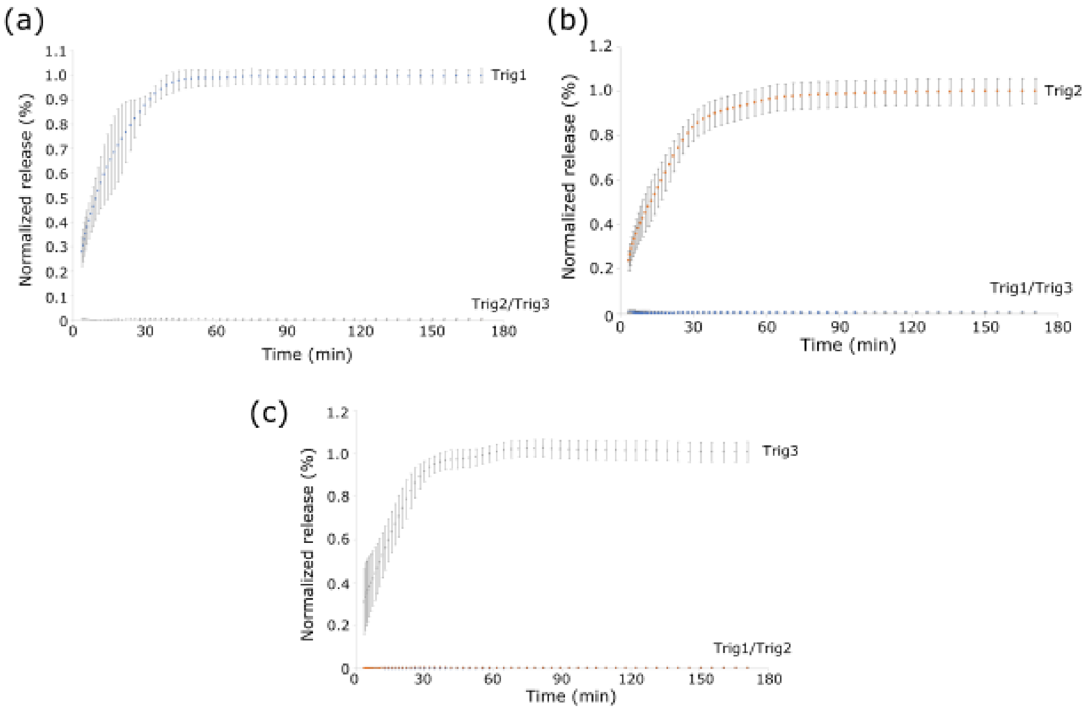

Figure S6. 4WJ Release Calibration within Solution. (**a**) 4WJ-HEX. The excitation for HEX is 540 nm and the emission was recorded at 570 nm. (**b**) 4WJ-FAM. The excitation for FAM is 465 nm and the emission was recorded at 510 nm. (**c**) 4WJ-TR. The excitation for TR is 585 nm and the emission was recorded at 618 nm**.**

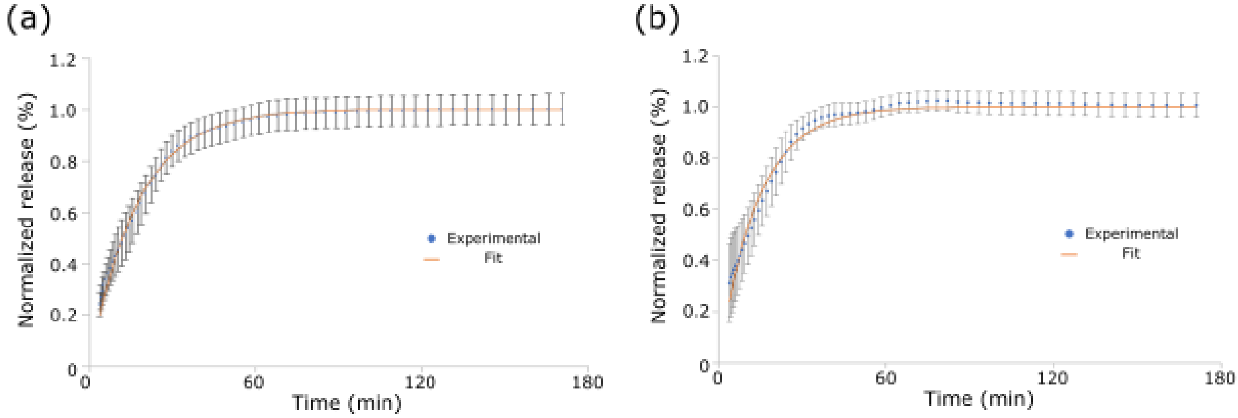

Figure S7. 4WJ release in solution (**a**) 4WJ-FAM. The excitation for FAM is 465 nm and the emission was recorded at 510 nm. (**b**) 4WJ-TR. The excitation for TR is 585 nm and the emission was recorded at 618 nm.

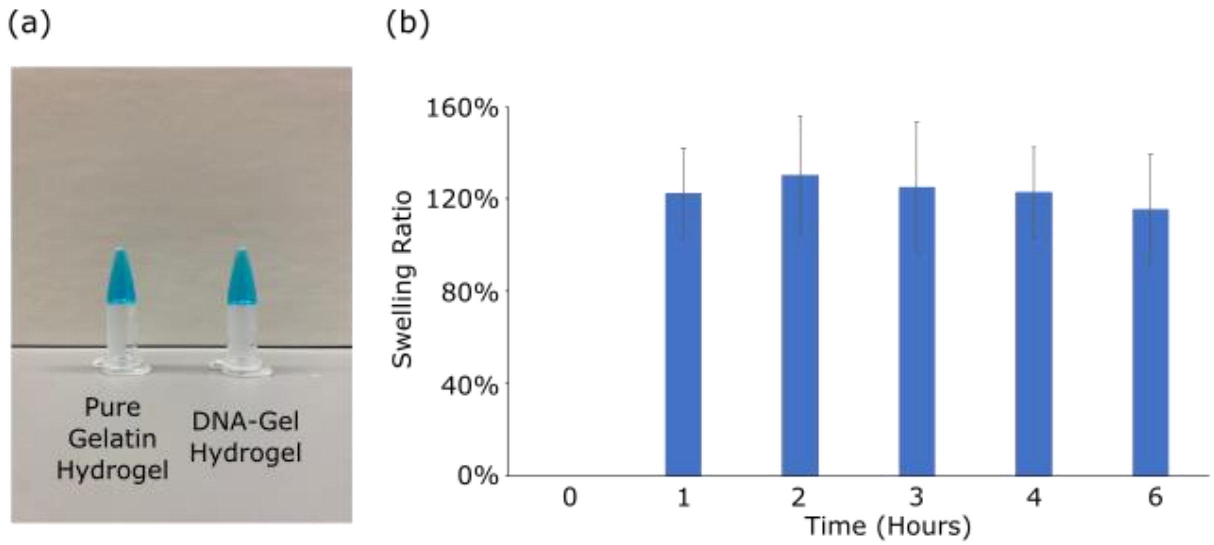

Figure S8. Hydrogel characterization (**a**) Image of both pure gelatin hydrogel and DNA-Gel hydrogel in a tube. Blue ink was added to better visualize the hydrogel within the tube. (**b**) Swelling ratio of the pure gelatin hydrogel in 1X PBS (pH 7.4) at room temperature over 6 h (n = 3).

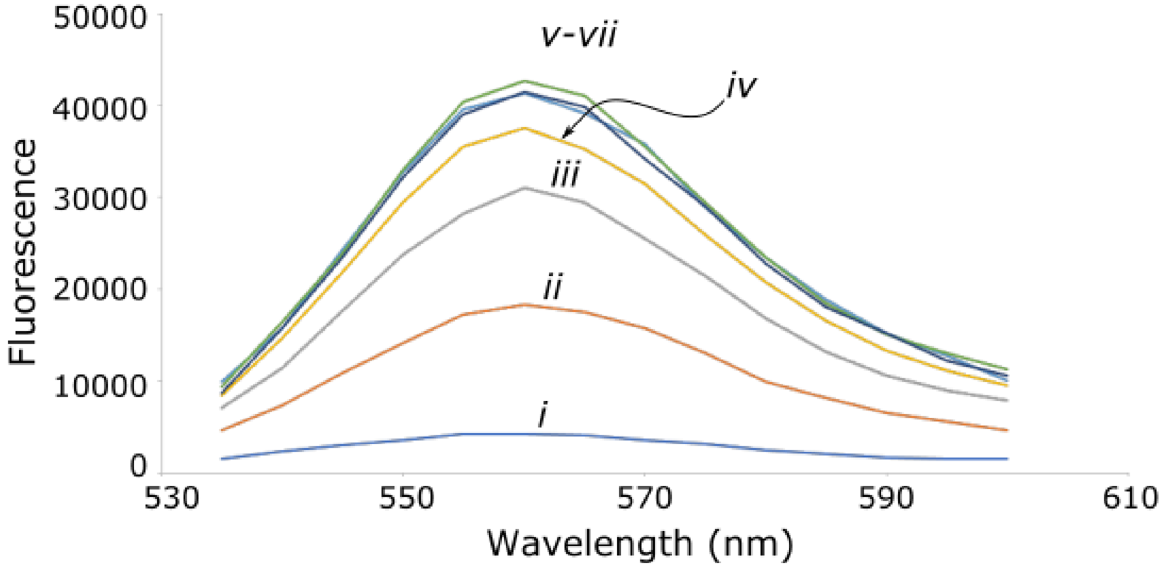

Figure S9. Representative fluorescence curves for the 4WJ-HEX release in DNA-Gel. (i) 0 h (ii) 1 h (iii) 3 h (iv) 5 h (v) 7 h (vi) 12 h (vii) 17 h.

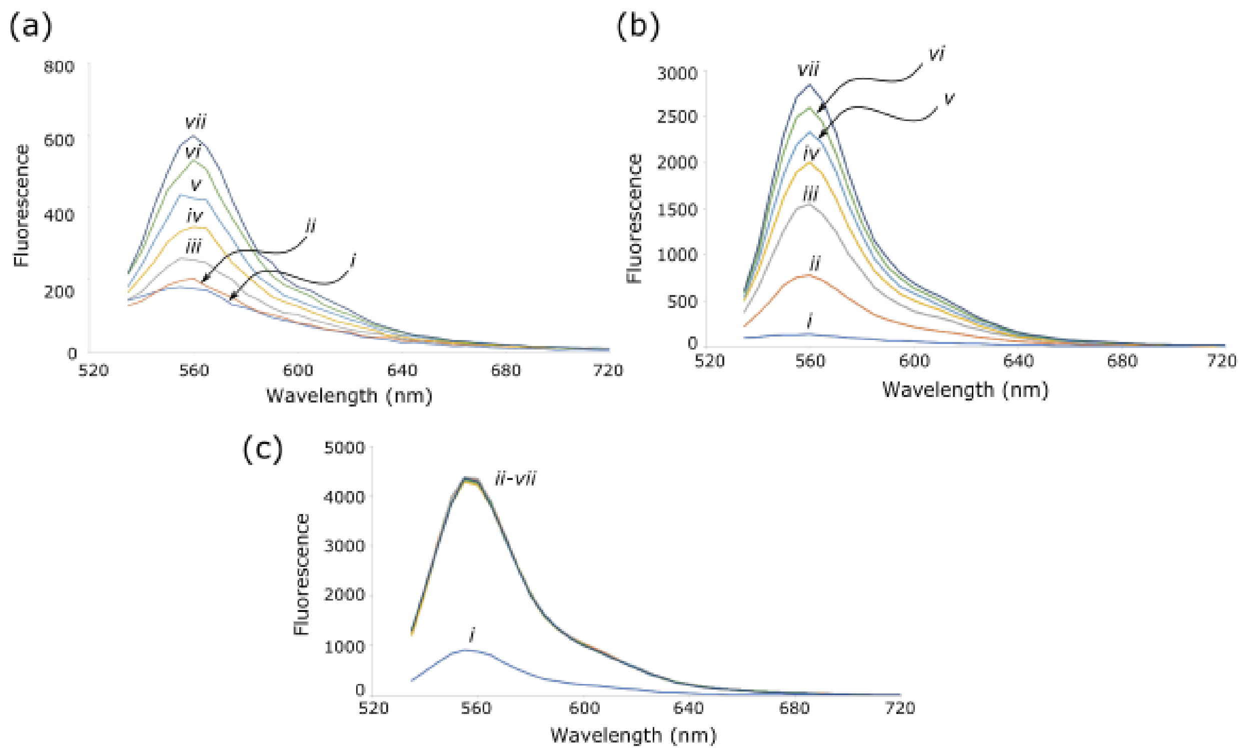

Figure S10. Representative fluorescence curves for the stability study for 4WJ-HEX (**a**) under serum condition in DNA-Gel hydrogel, (**b**) under DNAse condition in DNA-Gel hydrogel, and (**c**) under DNAse condition in solution. (i) 0 h (ii) 4 h (iii) 8 h (iv) 12 h (v) 16 hours (vi) 20 h (vii) 24 h.
